# The Possible Biotechnological Use of Edible Mushroom Bioproducts for Controlling Plant and Animal Parasitic Nematodes

**DOI:** 10.1155/2020/6078917

**Published:** 2020-06-24

**Authors:** Gloria Sarahi Castañeda-Ramírez, Juan Felipe de Jesús Torres-Acosta, José Ernesto Sánchez, Pedro Mendoza-de-Gives, Manases González-Cortázar, Alejandro Zamilpa, Laith Khalil Tawfeeq Al-Ani, Carlos Sandoval-Castro, Filippe Elias de Freitas Soares, Liliana Aguilar-Marcelino

**Affiliations:** ^1^Centro Nacional de Investigación Disciplinaria en Salud Animal e Inocuidad, INIFAP, Km 11 Carretera Federal Cuernavaca-Cuautla, No. 8534, Col. Progreso, Morelos, Jiutepec, CP 62550, Mexico; ^2^Facultad de Medicina Veterinaria y Zootecnia, Universidad Autónoma de Yucatán, Km 15.5 Carretera Mérida-Xmatkuil, CP 97100 Mérida, Yucatán, Mexico; ^3^El Colegio de la Frontera Sur, Apdo. Postal 36, C.P. 30700 Tapachula, Chiapas, Mexico; ^4^Centro de Investigaciones Biomédicas del Sur, Instituto Mexicano del Seguro Social, Argentina No. 1. Col. Centro, C.P. 62790 Xochitepec, Morelos, Mexico; ^5^School of Biology Science, Universiti Sains Malaysia, 11800 Minden, Pulau Pinang, Malaysia; ^6^Department of Plant Protection, College of Agriculture Engineering Science, University of Baghdad, Baghdad, Iraq; ^7^Department of Chemistry, Universidade Federal de Lavras, CEP, 37200900 Minas Gerais, Brazil

## Abstract

The present paper reviewed publications on the nematocidal activity of edible mushrooms (EM) and their potential use as sustainable tools for the control of parasitic nematodes affecting agriculture and livestock industry. Nematodes are organisms living in the soil and animals' guts where they may live as parasites severely affecting economically important crops and farm animals, thus causing economic losses to worldwide agriculture. Traditionally, parasitic nematodes have been controlled using commercial pesticides and anthelmintic (AH) drugs. Over the years, nematodes developed resistance to the AH drugs, reducing the usefulness of many commercial drugs. Also, the use of pesticides/anthelmintic drugs to control nematodes can have important negative impacts on the environment. Different EM have been not only used as food but also studied as alternative methods for controlling several diseases including parasitic nematodes. The present paper reviewed publications from the last decades about the nematocidal activity of EM and assessed their potential use as sustainable tools for the control of nematodes affecting agriculture and livestock industry. A reduced number of reports on the effect of EM against nematodes were found, and an even smaller number of reports regarding the potential AH activity of chemical compounds isolated from EM products were found. However, those studies have produced promising results that certainly deserve further investigation. It is concluded that EM, their fractions and extracts, and some compounds contained in them may have biotechnological application for the control of animal and plant parasitic nematodes.

## 1. Introduction

Traditionally, pesticides and anthelmintic (AH) drugs have been the most common methods for the control of parasitic nematodes affecting either crops or livestock [1, 2]. However, the misuse of those compounds has generated the presence of nematode strains highly resistant to most commercially available AH drugs [3]. Additionally, chemical residues of these compounds have generated soil and water pollution affecting beneficial organisms such as dung beetles, bacteria, fungi, soil worms, and mites [4]. Thus, the search for sustainable alternatives for the control of parasitic nematodes is gaining great interest worldwide [2, 5]. The use of nematophagous fungi as a biological control against parasitic nematodes is one of the alternatives that was extensively investigated [6] and now is being commercialized in many countries [7]. Meanwhile, a considerable body of research has investigated edible mushrooms (EM) for its diverse medicinal properties including anticancer, antimutagenic, antidiabetic, anti-inflammatory, antimicrobial, antibacterial, antifungal, antiviral, and antithrombotic [8–15]. However, the nematocidal activity of EM was only reported in the 1980s, particularly for different species of the *Pleurotus* genus [16]. After those early reports, other authors confirmed the ability of EM to produce nematocidal substances that immobilize nematodes, which are used by the fungi to complement their nutritional requirements [17, 18]. However, to this date, there is no review article summarizing the body of research produced in the last four decades on the nematocidal effects of EM. The search for the potential use of the nematocidal compounds from different EM species has included a small number of studies using free-living nematodes [19–25] and some studies exploring the activity against parasitic nematodes of plants [17, 20, 22, 26–29] under *in vitro* conditions. More recently, different EM materials began to be screened for their nematocidal activity against larval stages of ruminant parasitic nematodes [8, 30–37]. The present review confirmed that the number of scientific studies on the nematocidal activity of EM is still small. However, this review can help to visualize what has been done in the last four decades and what remains to be explored to be able to apply those materials for the control of parasitic nematodes of plants or animals. In this review, we generated a list of EM that has shown AH activity and the compounds or combination of compounds associated with that nematocidal activity. Furthermore, we identified the few published studies aiming to test EM for the control of nematodes in real-life conditions. Derived from the culturing process of EM, the search for nematocidal activity included different materials such as the mycelium, the degraded substrate containing mycelium (spent mushroom compost (SMC)), and the fruiting body itself. Fungi or fungus derivates possessing medicinal and nutritional properties for animals could also be investigated as nutraceutical products [38], which represent another alternative method for the control of parasitic nematodes [8]. The present paper reviewed publications in the last decades (1987-2020) reporting on the nematocidal activity of EM and their potential use as sustainable tools for the control of parasitic nematodes affecting agriculture and livestock industry.

## 2. Macromycetes

Macromycete mushrooms are filamentous organisms, lacking chlorophyll, saprobes, which can be visualized by the naked eye and take their nutrients from the organic matter where they grow. They reproduce asexually or sexually by means of spores. These fungi have a cell wall composed of chitin or cellulose, and their growth is apical. Macromycetes constitute a group of fungi that develop fruiting bodies.

This group of fungi may establish mutualistic relationships with tree roots called mycorrhizae, thus helping them in their functions [39]. In general, fungi are heterotrophic organisms; therefore, they depend on organic matter in decay; they can be saprobic, parasitic, or mutualistic; and they develop in different environments. This group of fungi can be classified as edible (i.e., *Pleurotus ostreatus*), poisonous (i.e., *Amanita abrupta*), and hallucinogenic (i.e., species of *Psilocybe*, *Stropharia*, and *Conocybe*) [40]. Its life cycle is complex and varies according to the genus of the fungus.

## 3. Edible Mushrooms

Although there is a great diversity of mushrooms, not all are edible, and only a few EM are cultivated commercially. The number of species of edible macromycetes that could be cultivated in the world varies from 92 to 130 species [41, 42] (Table 1). Several EM were used since ancient times, and they are appreciated for their taste and nutritional value [38]. It is worth mentioning that animals can also ingest this kind of mushrooms, either accidentally or naturally as part of their food; for example, they can feed on macromycetes that they find in the pastures [42]. However, many of them have not been studied in-depth to identify their potential medicinal properties. Thus, there is an ample opportunity to investigate the different EM aiming to find chemical compounds that could be used as alternative tools for the control of diseases, including parasitic nematodiasis.

Edible mushrooms are highly appreciated all over the world as they offer an important quantity and quality of nutrients for human consumption, while also contributing to the cure of many diseases. The nutritional quality of macromycete fungi has been reported in several studies. For example, the genus *Pleurotus* spp. has shown to be an important source of nutrients. Its ash content ranges from 76.6 to 87.9 g/kg [43], and its protein content is approximately 111 g/kg dry matter [44]. Besides, mushrooms of the genus *Pleurotus* spp. have a low-fat content, which is highly valued, and its fibre value ranges from 112.2 to 118.2 g/kg [43]; hence, it is also considered an important source of dietary fibre [45]. Likewise, its fat fraction includes substances such as triglycerides, phospholipids, steroids, free fatty acids, carotenoid pigments, and fat-soluble vitamins [46]. Also, macromycetes can produce different medicinal compounds that have been used by different cultures worldwide as a common practice [47] (Table 2).

### 3.1. Parasitic Nematodes of Importance in Agriculture and Livestock Industries

Parasitic nematodes may cause severe damage to animals and plants, resulting in great economic losses both in agriculture and livestock industries. Gastrointestinal nematodes (GIN) affecting animals, particularly those that affect small ruminants, such as *Haemonchus contortus*, can severely deteriorate animal health and in severe cases may cause the death of young animals. Infections with GIN are a common problem for grazing ruminants. While animals ingest grass and other plants, they may also consume nematode infective larvae, which climb towards the tip of leaves of grasses and herbs. Important economic losses have been reported from GIN infections in many countries. For example, in Mexico, yearly economic losses attributed to GIN have been recorded around $8,902 million Mexican pesos [1, 52], representing approximately $404 million USD (1 USD = 22 MXN).

Similarly, in the case of plants, there are phytoparasitic nematodes, which may cause significant losses, affecting around 200 different crops, including banana, chili, potato, and tomato [53]. Phytopathogenic nematodes may affect various crops in several countries including Argentina, Chile, Bolivia, Ecuador, Peru, the United States, and Mexico [2, 54]. Eighteen families with 84 plant species have been recognized as highly susceptible to the attack of phytopathogen nematodes [55], with the resulting economic losses.

Chemical treatments are currently used to fight animal and plant parasitic nematodes. However, the use of commercial chemical agents has triggered the problem of anthelmintic (AH) resistance in the parasite populations, substantially reducing the efficacy of treatments [3]. Additionally, animals treated with AH drugs eliminate chemical residues to the soil through defecation, causing an important impact on the environment. Soil contamination with AH drugs may affect beneficial organisms such as dung beetles, water fleas, earthworms, and nematophagous mites [2, 56]. Currently, alternative methods have been proposed to reduce the use of conventional AH drugs for the control of parasitic nematodes of agriculture and animal production [57]. These alternative methods should be used in an integrated manner to promote its sustainability [58].

### 3.2. Nutraceutical Materials as Tools for the Sustainable Control of Parasitic Nematodes

Many different plant species commonly used for their nutritional properties, either for humans or for animals, may also be used as nutraceuticals when they contain plant secondary compounds that could help control parasitic nematodes of the gastrointestinal tract [59]. The nutraceutical plant materials can be used as an alternative method for the control of GIN of ruminants [58]. Several studies performed in different countries have reported different plant materials with nutraceutical properties affecting GIN of ruminants [59–63]. Most of those studies used *in vitro* screening protocols to investigate different plant extracts or to identify bioactive molecules/metabolites with AH activity against ruminant parasitic nematodes [64, 65]. The protocols employed provide a useful guide to develop protocols to test EM and their derivatives. Early field studies with nutraceutical plants suggested that the ingestion of plants rich in condensed tannins and other secondary compounds reduced the excretion of GIN eggs in sheep faeces [66, 67]. Those field studies initiated the evaluation of different plants containing condensed tannins in temperate zones, e.g., *Onobrychis viciifolia* [68, 69]. Subsequently, the exploration of other plants, rich in tannins and other polyphenols, was initiated aiming to find other nutraceutical candidates showing *in vitro* and *in vivo* anthelmintic (AH) activity [57, 61]. The *in vitro* screening procedures showed that the activity of many tropical plant materials showing nutraceutical potential was associated with secondary compounds other than condensed tannins [70, 71]. Some of the bioactive compounds with AH activity were identified as terpenes and alkaloids [72–74]. Other compounds have shown nematocidal properties, i.e., caffeoyl and coumaroyl derivatives [75], as well as phenolic compounds and flavonoids [76, 77]. In the case of EM, the genus *Pleurotus* spp. has shown nematocidal activity, which has been attributed to a nematotoxin (as trans-2-decenedioic acid) [78]. However, recent *in vitro* studies using *H. contortus* eggs showed that the activity of the different bioactive compounds seems to work synergistically between two or more molecules, since the AH activity found for extracts and partitions containing different compounds was lost when the compounds were evaluated separately [64].

### 3.3. Activity of Edible Mushrooms against Different Nematode Species

It is known that fungi can complement their nitrogen nutritional requirements by feeding on nematodes [79]. According to [18], ten species of gilled fungi, including the oyster mushroom (*P. ostreatus*), have been shown to attack and consume nematodes. Some three decades ago, a study demonstrated the ability of *Pleurotus* spp. (*P. ostreatus*, *P. strigosus*, *P. subareolatus*, and *P. cornucopiae*) to destroy nematodes. Those fungi produced tiny droplets from structures in their mycelium, which supposedly contained a nematotoxin. When rhabditid nematodes touched those droplets, they suffered alterations in their head structure, caused the displacement of the oesophagus, and/or altered the tissues surrounding the oesophagus in less than a minute. Nematodes became immobilized, and the directional hyphae penetrated the body orifices, colonized, and digested the nematode [16]. Recent studies with *Pleurotus* spp. showed that the substances affecting nematodes were produced or present in specialized structures morphologically differentiated known as toxocysts [17, 79]. The toxin present in the toxocysts was identified as trans-2-decenoic acid [78]. The toxocysts are spherical structures pediceled and morphologically similar, which contain toxins and fatty acids that paralyze nematodes, allowing the adherence of the fungus to the nematode's sheath and degrading it to obtain nutrients [27]. The secretion of laccases has also been suggested as a mechanism of *Pleurotus* spp. to obtain nutritional resources from antagonistic nematodes present in their environment [79], but no evidence has been produced. Laccases are a part of a group of enzymes called polyphenol oxidases containing copper atoms, also called multicopper oxidases that oxidize polyphenols, aromatic diamines, and a range of other compounds [80].

### 3.4. Activity against Free-Living Nematodes

Free-living nematodes live in the soil where they play an important role in different ecological processes, i.e., food chains, nitrogen recycling, etc. [81]. These organisms are also used as biological markers, particularly as indicators of water pollution [82]. During the process of identifying fungal extracts and metabolites from EM with nematocidal activity, free-living nematodes have been used as a valuable model of study, representing one of the main groups of soil nematodes [83]. These nematodes are very useful for performing the initial screening and selection of EM products as potential candidates to be assessed against parasitic nematodes of plants or animals.

One of the first studies assessing the nematocidal activity of *P. ostreatus* extracts against free-living nematodes was published nearly 20 years ago [78]. In that study, the *in vitro* assessment of an aqueous extract obtained from *P. ostreatus* mycelia was tested against the free-living nematode *Panagrellus* sp. These authors reported 95% nematostatic activity, after 1 h of *in vitro* confrontation (96 wells), which was attributed to the effect of a compound identified as trans-2-decenedioic acid [78]. The *in vitro* nematocidal activity of *P. pulmonarius* and *Hericium coralloides* against the free-living nematode *Caenorhabditis elegans* was reported [19], and the chemical screening of *P. pulmonarius* resulted in various compounds with nematocidal activity, being the S-coriolic acid and linoleic acid the compounds with the highest activity, with an effective concentration 50 (EC_50_) between 5 and 10 ppm.

On the other hand, the mycelium of another EM, *Stropharia* sp., was also evaluated *in vitro* against the free-living nematode *P. redivivus* at different times of exposure, recording 100% mortality after 36 h exposure [20]. Similarly, *Panagrellus* sp. larvae were confronted *in vitro* with the mycelium of *P. ostreatus* mycelia, and 95% mortality was recorded at 72 h postconfrontation [21].

An aqueous extract from the fungus *P. eryngii* significantly reduced the number of *Panagrellus* sp. (60% and 90%), after 24 h and 48 h interaction, respectively [22]. Those authors emphasized that the nematocidal effect of the extract was not related to enzymatic activity (proteases), but the presence of other metabolites. On the other hand, acetonic and methanolic extracts obtained from the fruiting body of *P. ostreatus* were assessed *in vitro* against the nematode *P. redivivus*, during 1 h exposure. High nematode mortalities (80 and 92%) were recorded for the acetonic and methanolic extracts, at 75% (*v*/*v*), respectively [23]. More recently, Soares et al. [24] assessed an extract obtained from the SMC from another EM, *Hypsizygus marmoreus*, and they reported 52% reduction in the population of *P. redivivus* attributed to a protease activity produced by this extract. In addition, Ferreira et al. [25] demonstrated the nematocidal potential of *Flammulina velutipes* and from its SMC. The SMC, the isolated fungus, the crude extract, and the boiled crude extract reduced the *Panagrellus* sp. larva population. The SMC evidenced higher nematocidal activity (70%) than the isolated fungus (26%) after 72 h exposure. Moreover, the authors suggested that the nematocidal activity was due to proteolytic enzymes and other metabolites [25].

### 3.5. Activity of Bioproducts from Edible Mushrooms against Plant Parasitic Nematodes

One of the first reports showing the activity of EM against plant parasitic nematodes was published by Luo et al. [26]. In that study, the root-knot-nematode *Meloidogyne arenaria* was exposed to *Coprinus comatus* mycelia. The results showed that nematodes were paralyzed by 95.8% in an 8 h confrontation period. Likewise, the mycelium of *Stropharia* sp. was assessed against juveniles of the second stage (J_2_) of *M. incognita* [20]. Nematodes were exposed to the mycelia on potato dextrose agar (PDA) plates, and 100% mortality was recorded after 36 h postconfrontation. The mycelium of *P. eryngii* was assessed searching for ovicidal activity against *M. javanica* resulting in 53% ovicidal activity, which was attributed to fungal protease and chitinase action [22]. Meanwhile, the antagonistic effects of five species of *Pleurotus* (*P. ostreatus*, *P. sajor-caju*, *P. cornucopiae*, *P. florida*, and *P. eryngii*) against second-stage juveniles (J_2_) of *M. javanica* were studied, *in vitro.* All the species tested produced tiny droplets of a toxin [17]. Nematodes touching such droplets recoiled immediately, became inactive, and were colonized by the fungi after 24-48 h particularly through their mouth. These effects were more evident for *P. ostreatus*. Filtrates of the tested fungi grown in malt extract broth were toxic on the nematodes, but the filtrates of *P. ostreatus* showed the highest nematocidal activity against *M. javanica* J_2_. Likewise, in Japan, the mycelium of the fungus *P. cystidiosus* was studied and showed to produce toxocysts in the presence of pinewood nematode *Bursaphelenchus xylophilus* [27]. In another study, an acetonic fraction obtained from *P. ferulae* mycelia was evaluated in *in vitro* searching for nematocidal activity and potential nematocidal compounds against the wood-affecting nematode *B. xylophilus.* The activity observed was associated with a major compound identified as 5-hydroxymethyl-furancarbaldehyde with an EC_50_ of 54.7 mg L^−1^ [28]. The ethanolic and aqueous extract of *P. ostreatus* was also evaluated against *Meloidogyne* spp. nematodes, finding 99.1% mortality for the ethanolic extract and 1.4% for the aqueous extract after 60 min [36]. On the other hand, extracts obtained from the fruiting body of *P. ostreatus* were assessed *in vitro* against the nematode *Ditylenchus dipsaci*, during 1 h exposure. High nematode mortalities (95%) were recorded for the acetonic and methanolic extracts, at 84% (*v*/*v*), respectively [23]. Finally, in a recent study, the aqueous extracts obtained from fruiting bodies of a number of EM, including *Amanita muscaria*, *Boletus* sp., *Lactarius deliciosus*, *P. citrinopileatus*, *P. ostreatoroseus*, *P. ostreatus*, *P. pulmonarius*, *P. sajor-caju*, *Russula amethystina*, and *Suillus* sp., were investigated to identify possible nematocidal activity against *M. incognita* [29]. After a 24 h *in vitro* confrontation, all mushroom extracts showed high nematocidal activities with mortality percentages reaching 90.7 to 100.0% [29].

### 3.6. Activity of Bioproducts from Edible Mushrooms against Animal Parasitic Nematodes

#### 3.6.1. *In Vitro* Studies

There are only a few studies evaluating the *in vitro* nematocidal activity of products obtained from EM against ruminant parasitic nematodes [8, 30, 32, 33]. A study performed in Denmark showed that *P. pulmonarius* mycelium had an immobilizing effect against preinfective larval populations of *Ostertagia ostertagi*, *Cooperia oncophora*, *Oesophagostomum quadrispinulatum*, and *Cyathostoma* sp. The fungus showed a stronger immobilization effect against the preinfective stages (70%) when compared to the infective larvae (30%) [34].

In an experiment performed in Mexico, mycelia of a group of EM strains were assessed. The results showed a range of mortality from 76.3 to 93.9% for several species including *Pleurotus ostreatus*, *P. eryngii*, *P. cornucopiae*, and *Lentinula edodes*. Meanwhile, the activity for *Coprinus comatus*, *L. boryanus*, and *Panus* sp. ranged from 10.03 to 56.3% mortality [30]. In another study, the SMC of *P. ostreatus* consisting in corn pod, Pangola grass, sawdust, and coffee pulp was processed to obtain a hydroalcoholic extract which was assessed for its potential activity against *H. contortus* eggs. The results showed 99.3% egg hatching inhibition (EHI) at 0.5 mg/mL after 72 h exposure [31]. Likewise, a bioguided study using a hydroalcoholic extract obtained from *P. ostreatus* mycelia was performed, and one fraction from this extract caused 100% EHI at 1.25 mg/mL after 72 h exposure. The authors also reported several metabolites present in the bioactive fraction, including four fatty acids: (i) hexadecanoic acid, (ii) octadecanoic acid, (iii) 2-butoxy phosphate ethanol, and (iv) 2-butoxy phosphate ethanol (3 : 1) and a xylitol sugar [35]. In another study, Vieira et al. [32] evaluated aqueous extracts obtained from *Agaricus blazei* fruiting bodies against *H. contortus* eggs and found 100% EHI at 3.62 mg/mL after 72 h confrontation. Similarly, a hydroalcoholic extract from *P. djamor* fruiting bodies was assessed against eggs and infective larvae of *H. contortus*, and one of the fractions obtained from this extract showed 100% EHI at 10 mg/mL after 72 h exposure. This fraction also showed 90.6% larvicidal activity at 40 mg/mL after 72 h exposure. The compounds identified in the bioactive fraction were 4 fatty acids: (i) pentadecanoic, (ii) hexadecanoic, (iii) octadecadienoic, and (iv) octadecanoic acid and a terpene identified as *β*-sitosterol. This fraction showed to be active against eggs of *H. contortus* [8]. Another study evaluated the *in vitro* nematocidal activity of ethanolic extracts from seven strains of *P. eryngii* against *H. contortus* eggs and larvae (L_3_) [33]. These extracts caused low larval mortality (11.55 to 18.83%) at a concentration of 20 *μ*g/mL. However, when an extract of *P. eryngii* (ECS-1255 strain) was fractionated (F1 to F5 fractions), a high ovicidal activity 91.87% at 40 mg/mL was found with F5. The GC-MS analysis of this fraction showed the presence of trehalose, polyols (L-iditol, galactitol, D-mannitol, D-glucitol, and myoinositol), adipic acid, stearic acid, squalene, and *β*-sitosterol, which could be responsible for the activity [33]. A recent study evaluated the aqueous and ethanolic extracts obtained from *P. ostreatus* mycelium against a rabbit parasitic nematode species (*Passalurus* sp.) at concentrations of 75% *v*/*v* of the ethanolic extract with 10, 20, 30, and 60 min of exposure. Nematode mortality was 99.10% at 60 min [36]. More recently, an extract obtained from the SMC of *H. marmoreus* was evaluated and reported a 26% reduction in the bovine larval population [24]. Finally, pure molecules (pentadecanoic acid, palmitic acid, *β*-sitosterol, stearic acid, and linoleic acid) previously reported in the fungi were evaluated against *H. contortus* eggs and larvae [8]. Palmitic and stearic acids inhibited hatching by 100% at 20 mg/mL. However, the combination of the two compounds showed hatching inhibition of 70 to 100%. On the other hand, in the larval mortality test, the combination of the five compounds showed 100% mortality at 20 mg/mL at 24 h. Thus, the activity found with the combination of molecules present in the edible fungi suggests synergistic activity [37].

#### 3.6.2. *In Vivo* Studies

In our review, only one report was found on the potential AH effect of EM consumption by sheep [32]. This report evaluated the consumption of the *Agaricus blazei* fruiting body against *H. contortus* at 11.4 g/kg LW for 2 consecutive days in lambs. A reduction of eggs per gram of faeces (EPG) was initially observed with respect to the elimination of EPG in untreated animals. After 14-day consumption, the EPG reduction was not significant [32].

### 3.7. Metabolites with Anthelmintic/Nematocidal Activity

Since several antibiotics were derived from fungi, the search for secondary metabolites with antiparasitic activity possesses special importance for agriculture and livestock industries. Thus, the evaluation of the AH activity of the fungi and their molecules responsible of this activity could lead to finding new molecules that could replace AH drugs currently used (Table 3).

The use of EM and their products (mycelium, fruiting bodies, and degraded substrate) for the control of parasitic nematodes of livestock has not been widely explored. Furthermore, it is worth mentioning that agroindustrial residues derived from the EM cultivation are currently considered just a waste. To take advantage of the latter for a beneficial purpose, it is important to explore their potential application as a natural source of AH compounds that could become valuable tools for the control of parasitic nematodes affecting agriculture and livestock industry.

## 4. Conclusions

Most studied EM species with reported nematocidal activity belong to the genus *Pleurotus.* The AH activity has been reported for extracts and its fractions obtained from fruiting bodies, mycelium, and degraded substrate. Among the bioactive molecules reported in the mushroom extracts with nematocidal activity, there are fatty acids such as pentadecanoic acid, hexadecanoic acid, octadecadienoic acid, and octadecanoic acid and a terpene *β*-sitosterol. Only one study recorded the *in vivo* nematocidal activity of *Agaricus blazei* fruiting body against *H. contortus* in sheep. Further studies on the potential use of products obtained from fruiting bodies, mycelia, and SMC should be encouraged to establish whether these materials can be considered as natural control methods for the benefit of agriculture worldwide.

## Figures and Tables

**Table 1:** Edible macromycete fungus species with potential to be cultivated for human consumption [41].

*Agaricus arvensis*, *A. augustus*, *A. bisporus*, *A. bitorquis*, *A. blazei*, *A. brunnescens*, *A. campestres*, *A. cylindracea*, *A. fuscosuccinea*, *A. molesta*, *A. polytricha*, *A. praecox*, *A. subrufescens*, *Agrocybe aegerita*, *Albatrellus spp.*, *Armillaria mellea*, *Auricularia auricula-judae*	*Calvatia gigantea*, *Coprinus comatus*, *Daedalea quercina*, *Dictyophora duplicata*, *Flammulina velutipes*, *Fomes fomentarius*, *Ganoderma applanatum*, *G. curtisii*, *G. lucidum*, *G. oregonense*, *G. sinense*, *G. tenus*, *G. tsugae*, *Grifola frondosa*, *Hericium coralloides*, *H. erinaceus*	*Hypholoma capnoides*, *H. sublateritium*, *Hypsizygus marmoreus*, *H. tessulatus*, *Inonotus obliquus*, *Kuehneromyces mutabilis*, *Laetiporus sulphureus*, *Laricifomes officinalis* (=*Fomitopsis officinalis*), *Lentinula edodes*, *Lentinus strigosus* (*Panus rudis*), *Lentinus tigrinus*	*Lentinus tuber-regium*, *Lepista nuda*, *L. sórdida*, *Lyophyllum fumosum*, *L. ulmarium* (*=Hypsizygusulmarium*), *Macrocybe gigantea* (*=Tricholoma giganteum*), *Macrolepiota procera*, *Marasmius oreades*, *Morchella angusticeps*, *M. esculenta*, *Neolentinus lepideus* (=*Lentinus lepideus*)	*Oligosporus spp.*, *Oudemansiella radicata*, *Oxysporus nobilissimus*, *Panellus serotinus* (=*Hohenbuehelia serotina*), *Paneolus subbalteatus*, *P. tropicalis*, *Phallus impudicus*, *Phellinus spp.*, *Pholiota nameko*, *Piptoporus betulinus*, *P. indigenus*, *Pleurocybella porrigens*, *Pleurotus citrinopileatus*, *P. cornucopiae*, *P. cystidiosus*, *P. djamor*, *P. eryngii*, *P. euosmus*, *P. ostreatus*, *P. pulmonarius*, *P. rhodophillus*	*Pluteus cervinus*, *Polyporus indigenus*, *P. saporema*, *P. umbellatus* (=*Dendropolyporus umbellatus*), *Psilocybe cyanescens*, *Schyzophyllum commune*, *Sparassis crispa*, *Stropharia rugosoannulata*, *Trametes cinnabarinum*, *T. versicolor*, *Tremella fuciformis*, *Volvariella bombycina*, *V. volvácea*, *V. volvacea var. Gloiocephala*

**Table 2:** Medicinal effect and bioactive compounds identified from edible mushrooms [47].

Fungal species	Medicinal effect	Active compound	Reference
*Agaricus bisporus*	Antioxidant	Phenolic compounds	[48]
		Flavonoids	
		B-carotenes	

*Boletus edulis*	Antioxidant	Phenolic compounds	[48]
		Flavonoids	
		B-carotenes	

*Pleurotus spp.*	Antiviral		[49]
	Antibiotic	Polysaccharides	

*Pleurotus ostreatus*	Antioxidant	Phenolic compounds	[48, 49]
	Antibiotic	Flavonoids	
	Antibacterial	B-carotenes	
	Antihumoral	Polysaccharides	
		B-D glucan	
		Glycopeptides	

*Lactarius deliciosus*	Antibacterial	Sesquiterpenes	[50]

*Lactarius indigo*	Antihumoral	Organic extracts	[50]
	Anti-inflammatory	Terpenoids	
		Polyphenols	

*Ramaria botrytis*	Antioxidant	Phenolic compounds	[51]
		Tocopherol	
		Carotenoids	
		Ascorbic acid	

**Table 3:** Edible mushrooms reported with nematocidal activity against nematodes belonging to different taxa.

Mushroom species	Blank nematode	Stage	Bioactive molecule	Author
*P. cornucopiae*, *P. cystidiosus*, *P. ostreatus*, *P. strigosus*, *P. subareolatus*	Rhabditoid	Larvae	Not reported	Thorn and Barron, 1984 [18]
*P. ostreatus*, *P. strigosus*, *P. subareolatus*, and *P. cornucopiae*	Rhabditoid	Larvae	Not reported	Barron and Thorn, 1987 [16]
*P. pulmonarius*	*Ostertagia ostertagi*, *Cooperia oncophora*, *Oesophagostomum quadrispinulatum Cyathostoma sp.*	Preinfective larvae	Not reported	Larsen and Nansen, 1991 [34]
*P. ostreatus*	*Panagrellus sp.*	Larvae	Trans-2-decenedioic acid	Kwok et al., 1992 [78]
*P. pulmonarius*	*Caenorhabditis elegans*	Larvae	S-coriolic acid (1), linoleic acid (2), p-anisaldehyde (3), p-anisyl alcohol (4), 1-(4-methoxyphenyl)-1,2-propanediol (5), and 2-hydroxy-(4′-methoxy)-propiophenone (6)	Stadler et al., 1994 [19]
*Coprinus comatus*	*Meloidogyne arenaria*	Juveniles (J_2_)	Not reported	Luo et al., 2004 [26]
*P. ostreatus*, *P. sajor-caju*, *P. cornucopiae*, *P. florida*, and *P. eryngii*	*M. javanica*	Juveniles (J_2_)	Not reported	Heydari et al., 2006
*P. ferulae*	*Bursaphelenchus xylophilus*	Not identified	Cheimonophyllon E; 5*α*,8*α*-epidioxyergosta-6,22-dien-3*β*-ol; 5-hydroxymethyl-furancarbaldehyde	Li et al., 2007 [28]
*P. cystidiosus*	*Bursaphelenchus xylophilus*		Not reported	Truong et al., 2007 [27]
*Stropharia* sp.	*Panagrellus redivivus*	Larvae	Not reported	Chuixu et al., 2013 [20]
*Stropharia* sp.	*Meloidogyne incognita*	Larvae	Not reported	Chuixu et al., 2013 [20]
*P. ostreatus*, *P. eryngii*, *P. cornucopiae*, *Coprinus comatus*, *Panus* sp., *Lentinula edodes*, and *L. boryanus*	*Haemonchus contortus*	Larvae	Not reported	Comans-Pérez et al., 2014 [30]
*P. ostreatus*	*Panagrellus sp.*	Larvae	Not reported	Hugo et al., 2015 [21]
*P. ostreatus*	*H. contortus*	Eggs	Not reported	Díaz, 2015 [31]
*P. ostreatus*	*H. contortus*	Larvae and eggs	Xylitol, hexadecanoic acid, octadecanoic acid, 2-butoxy phosphate ethanol, 2-butoxy phosphate ethanol (3 : 1), and a xylitol sugar	Cedillo, 2016 [35]
*P. djamor*	*H. contortus*	Larvae and eggs	Fraction E1: (i) pentadecanoic, (ii) hexadecanoic, (iii) octadecadienoic, and (iv) octadecanoic acid and a terpene identified as *β*-sitosterol	Pineda-Alegría et al., 2017 [8]
*Agaricus blazei*	*H. contortus*	Eggs	Not reported	Vieira et al., 2017 [32]
*P. eryngii*	*Panagrellus sp.*	Larvae	Not reported	Sufiate et al., 2017 [22]
*P. eryngii*	*Meloidogyne javanica*	Eggs	Proteases and chitinases	Sufiate et al., 2017 [22]
*P. eryngii*	*H. contortus*	Eggs and larvae	Fraction E5: trehalose, polyols (L-iditol, galactitol, D-mannitol, D-glucitol, and myoinositol), adipic acid, stearic acid, squalene, and *β*-sitosterol	Cruz-Arevalo et al., 2018 [33]
*P. ostreatus*	*P. redivivus*	Larvae	Not reported	Aldaz-Merchán, 2018 [23]
*P. ostreatus*	*Ditylenchus dipsaci*	Larvae	Not reported	Aldaz-Merchán, 2018 [23]
*P. ostreatus*	*Passalurus sp.*	Larvae	Not reported	Alvear-Díaz, 2018 [36]
*P. ostreatus*	*Meloidogyne sp.*	Larvae	Not reported	Alvear-Díaz, 2018 [36]
*P. ostreatus*, *Boletus* sp., *P. pulmonarius*, *P. citrinopileatus*, *A. muscaria*, *R. amethystina*, *L. deliciosus*, *Suillus* sp., *P. sajor-caju*, and *P. ostreatoroseus*	*M. incognita*	Larvae J_2_	Not reported	Wille et al., 2019 [29]	
*Hypsizygus marmoreus*	*P. redivivus*	Larvae	Proteases	Soares et al., 2019 [24]
*Hypsizygus marmoreus*	*Haemonchus* spp., *Cooperia* spp., and *Oesophagostomum* spp.	Larvae	Proteases	Soares et al., 2019 [24]
*Flammulina velutipes*	*Panagrellus* sp.	Larvae	Enzymes and metabolites	Ferreira et al., 2019 [25]
*Pleurotus djamor*	*H. contortus*	Larvae and egg	Pentadecanoic acid, palmitic acid, *β*-sitosterol, stearic acid, and linoleic acid	Pineda-Alegría et al., 2020 [37]
